# Effects of Dietary Anise (*Pimpinella anisum* L.) Oil on Growth Performance, Blood Parameters and Muscle Nutrient Content of Nile Tilapia (*Oreochromis niloticus*)

**DOI:** 10.1002/vms3.70443

**Published:** 2025-06-02

**Authors:** Serkan Tok, Mustafa ÖZ, Suat Dikel

**Affiliations:** ^1^ Department of Fisheries and Diseases Graduate School of Health Sciences, Aksaray University Aksaray Türkiye; ^2^ Department of Fisheries and Diseases Faculty of Veterinary Medicine, Aksaray University Aksaray Türkiye; ^3^ Department of Aquaculture Faculty of Fisheries, Cukurova University Adana Türkiye

**Keywords:** anise oil, antioxidant properties, blood parameters, growth performance, Nile tilapia

## Abstract

**Objectives:**

This study aimed to investigate the effects of dietary *Pimpinella anisum* (anise) oil supplementation on growth performance, haematological and biochemical parameters, antioxidant enzyme activities and tissue histopathology in Nile tilapia (*Oreochromis niloticus*). The goal was to determine the optimal inclusion level that promotes health benefits without inducing toxicity.

**Methodology:**

A total of 240 juvenile Nile tilapia were randomly distributed into four groups: control (0.00%) and three treatments receiving anise oil at 0.10%, 0.20% and 0.30% for 8 weeks. Growth performance (weight gain, feed conversion ratio), haematological (RBC, Hb, WBC) and biochemical parameters (ALT, AST, glucose, lipids), oxidative stress markers (SOD, CAT, GPx) and histopathological changes in gill, liver, kidney and muscle tissues were evaluated.

**Results:**

Anise oil supplementation at 0.10% and 0.20% significantly improved growth performance, haematological indices and antioxidant enzyme activities (*p* < 0.05) compared to the control group. However, fish fed 0.30% anise oil showed elevated liver enzymes, glucose and lipid levels, along with moderate tissue damage, indicating potential adverse effects at higher doses.

**Conclusions:**

Dietary inclusion of 0.10% to 0.20% anise oil enhances the growth, physiological health and antioxidant defence in Nile tilapia. Nevertheless, 0.30% inclusion may impair metabolic balance and tissue integrity. Anise oil represents a promising natural feed additive for sustainable aquaculture when used at optimal levels.

## Graphical abstract

This study investigates the dietary inclusion of anise oil (*Pimpinella anisum* L.) in Nile tilapia (*Oreochromis niloticus*) feed and its impacts on growth performance, haematological indices and muscle nutrient composition. Supplementation with 0.10%–0.20% anise oil significantly enhanced fish growth, improved blood health indicators and increased muscle protein and lipid content. However, excessive inclusion (0.30%) induced oxidative stress and histopathological alterations. These findings highlight the functional potential of anise oil as a natural feed additive for sustainable tilapia aquaculture.

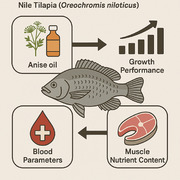

## 1 Introduction

With the growing awareness of consumers, the demand for aquaculture products continues to rise steadily. As a rapidly expanding sector, aquaculture has become one of the primary contributors to global food production, playing a vital role in ensuring food security worldwide. In recent years, the aquaculture industry has supplied more than half of the fish consumed globally, a trend that is expected to continue as production levels increase further to meet the rising demand (FAO 2023).

Nile tilapia (*Oreochromis niloticus*) is one of the most widely farmed freshwater fish species globally due to its rapid growth, high marketability, adaptability to diverse environmental conditions and ease of fry production. Its increasing importance in global aquaculture stems from both economic value and low nutritional requirements, making it a favourable candidate for sustainable and cost‐effective production systems (Ashouri et al. 2023; Bonham 2022). However, tilapia production faces some challenges, such as high feed costs, environmental impact and reproductive control. Therefore, it is necessary to find alternative feed ingredients and additives for tilapia farming (Öz et al. 2024). The successful cultivation of fish in intensive aquaculture systems depends on the availability of high‐quality and functional feeds that support optimal growth and health (Paray et al. 2021). Consequently, developing sustainable feeding strategies is essential to ensure the economic viability and environmental responsibility of modern aquaculture practices (Öz 2025). One effective approach is the use of feed additives, which are incorporated into fish diets to enhance productivity and improve the overall well‐being of aquatic species (Dawood et al. 2018).

Among commercially significant fish species, Nile tilapia (*O. niloticus*) have been successfully introduced in various aquaculture and fisheries programmes worldwide (El‐Sayed and Fitzsimmons 2023). It is widely regarded as one of the most commercially valuable species in global aquaculture, ranking as the second most farmed fish species in several regions, including Taiwan (Liu et al. 2022). The importance of Nile tilapia in aquaculture and experimental studies is evident from its wide range of uses, economic value, environmental impacts, genetic diversity and potential applications in food nutrition. Therefore, it is critical that research and experimental work on Nile tilapia continue in order to ensure sustainable aquaculture practices and broader scientific understanding.

In recent years, scientists working in the field of fish nutrition have strongly recommended the use of herbal feed additives (İnanan and Acar 2019; Limbu et al. 2021; Öz and Dikel 2022; Dikel 2015; Inanan et al. 2021). In addition, herbal products are used for their antimicrobial effects, enhancing growth performance and preventing stress that may occur in intensive production environments (Harikrishnan et al. 2011).

Anise (*Pimpinella anisum* L.) is an aromatic herb classified under the Apiaceae family (Akbar and Akbar 2020; Nasır and Yabalak 2021). It thrives in warm climates and is widely cultivated in various tropical and subtropical regions, including Southeast Asia and Southern Europe (W. Sun et al. 2019). The seeds of anise are rich in bioactive compounds, including phenolic acids, essential oils, eugenol, anisaldehyde, trans‐anethole, coumarins, polyacetylenes, polyenes and estragole (W. Sun et al. 2019). Due to its diverse biochemical composition, anise is recognized for its growth‐promoting, antibacterial, immunostimulatory and antioxidative properties (Yazdi et al. 2014). Beyond its application in aquaculture, anise has been extensively studied in human medicine, where it has demonstrated anticancer, antitumor, antiplatelet, hypolipidemic, anti‐inflammatory and antihaemolytic properties (Yu et al. 2021). Additionally, it has been effectively incorporated into broiler chicken diets, where it has shown the ability to enhance growth performance, feed conversion efficiency, immune response, antibacterial resistance and antioxidative status (Al‐Shammari et al. 2017; Gupta et al. 2019; Yazdi et al. 2014).

The use of aniseed in aquaculture remains limited; however, further research is warranted to explore its potential benefits on fish growth, health and overall welfare. In previous studies, dietary supplementation with aniseed in common carp (*Cyprinus carpio*) has been associated with enhanced growth performance, improved haematological parameters and increased antibacterial activity (Al‐Ashaab et al. 2017; Areej et al. 2012). These findings suggest that aniseed may serve as a valuable functional feed additive in aquaculture, but additional investigations are necessary to fully understand its impact across different fish species and production systems. It has been reported that aniseed added at a rate of 2.5%–3.5% to European sea bass feeds increases the welfare of the fish and can, therefore, increase growth performance and feed utilization and improve blood parameters (Ashry et al. 2022). It has been reported that aniseed can increase growth performance and support the immune system in rainbow trout when used at appropriate times and doses, but high doses may cause negative effects on haematological, serological and immunological parameters (Çelik 2021).

Although there are some studies on the use of anise oil in aquaculture feeds, there is no information in the literature on its use in Nile tilapia feeds. Therefore, this study aims to evaluate the effects of pure anise oil on growth performance, muscle nutrient content, histopathology, haematological and some biochemical parameters and oxidative stress parameters of Nile tilapia.

## 2 Material and Method

### 2.1 Fish Material and Experimental Design

This study was conducted within the framework of the ethical approval obtained from the Local Ethics Committee on 20 July 2023 and in accordance with the principles of the Local Ethics Committee. In the study, 120 fish with an average initial body weight of 74.20 ± 1.24 g were used. The research was carried out in 80‐L aquariums with 10 fish each in 3 replicates and 30 fish were used for each group. In order to maintain a consistent water temperature across all experimental groups during the duration of the study, an Eheim brand 100‐watt thermostat heater was utilized, ensuring that the water temperature remained stable at 25°C. Throughout the duration of the investigation, the fish in the experiment were nourished with tilapia feed manufactured by a commercial brand. The anise oil used in the experiment was obtained from a commercial company (Alfasol; TR‐34‐K‐044015). Anise oil was added to fish feeds by spraying at the rates of 0.00%, 0.10%, 0.20% and 0.30%. Anise oil inclusion rates were determined based on previous studies (Ashry et al. 2022). The fish feeds were prepared in 100 g batches.

### 2.2 Feeding Protocol and Method

The fish brought to the research unit were weighed at the end of the 15‐day adaptation period, and the research was started by taking the averages. The fish were fed two meals daily at 08:30 AM and 5:00 PM for 30 days. Water temperatures were checked in the morning and evening with digital thermometers placed in the aquariums. The airstones were cleaned at least twice a week for regular distribution of the air coming from the air motors, and the oxygen content of the water was measured once a day using an OxyGuard brand oxygen meter after feeding. The average weights of the fish were taken twice during the feeding period, and the experiment was completed at the end of Day 40.

### 2.3 Measurement of Fish Growth Parameters

After the feeding period was completed, the length and weight of the harvested fish were measured meticulously. Using the measurement results obtained as a result of the research, Specific Growth Rate (SGR) (Company et al. 1999), Feed Conversion Ratio (FCR) (Santinha et al. 1999), Protein Efficiency Ratio (PER) (Skalli and Robin 2004), the Condition Factor (CF) (Arellano‐Martínez and Ceballos‐Vázquez 2001) and the Hepatosomatic Index (HSI) (%) (Cheng et al. 2006) were calculated.

### 2.4 Blood Sampling and Analysis

Upon completion of the feeding experiment, the fish were subjected to anaesthesia using a concentration of 300 ppm of 2‐phenoxyethanol. Subsequently, the fish were promptly cleaned with a solution of 70% ethanol. Following the cleaning process, blood samples were collected from the vena caudalis using syringes that were pre‐treated with heparin. For haematological analysis, the blood sample was separated into conventional lavender‐top blood collection tubes containing anticoagulant (EDTA). Additionally, standard red‐top (SST II) advance serum separator tubes were used to analyse serum biochemical characteristics. The samples underwent centrifugation at a speed of 13,000 × *g* at a temperature of 4°C for a duration of 10 min in order to get serum. The haematological parameters were assessed promptly, while the serums were preserved at a temperature of −80°C until the biochemical parameters could be analysed. WBCs were counted using a counting chamber. The haematology autoanalyser MS4‐S (Melet Schloesing Laboratories, Osny, France) was used to analyse red blood cells (RBCs), mean cell volume (MCV), mean cell haemoglobin (MCH), MCHC, haematocrit (Hct) and haemoglobin (Hb). A manual haematological examination, following the method of Blaxhall and Daisley, was conducted on all blood samples collected in K3EDTA tubes to verify the accuracy of the automated blood count equipment results (Blaxhall and Daisley 1973).

### 2.5 Determination of Biochemistry Parameters

Alkaline phosphatase (ALP), aspartate aminotransferase (AST), alanine aminotransferase (ALT), total protein (TP), triglyceride (Trg), cholesterol (Cho) and Glucose (Glu) values were examined from the extracted blood serum. An automatic biochemistry device (MINDRAY‐BS400) was used for the analysis of biochemical parameters.

### 2.6 Oxidative Stress Parameters

Sera extracted from the blood samples of fish were analysed for total antioxidant status (TAS), total oxidant status (TOS), superoxide dismutase (SOD), Catalase (CAT), glutathione peroxidase (GPx) and malondialdehyde (MDA). TAS levels were measured using Relassay (Cat no.: RL0017) commercial kits (Erel 2004). TOS levels were evaluated using Relassay (Cat no.: RL0024) commercial kits (Erel 2005). The ratio of TOS to TAS is considered an oxidative stress index (OSI). The TAS unit obtained for calculation was converted to µmol/L, and the OSI value was calculated according to the following formula.

OSI = TOS (µmol H_2_O_2_ equivalent/L)/TAS (µmol Trolox equivalent/L) (Yumru et al. 2009; Kosecik et al. 2005; Harma et al. 2003).

MDA level, a product of lipid peroxidation, was determined according to Alak et al. (2020). SOD enzyme activity was determined by spectrophotometer (560 nm) according to the NBT (nitro blue tetrazolium chloride) reduction method with O_2_ under light (Y. I. Sun et al. 1988). Measurement of CAT activity: samples were mixed with 1 mL H_2_O_2_ (50 mM) and reacted at 37°C for 1 min. Then, 1 mL of ammonium molybdate was added to terminate the reaction, resulting in the formation of a yellowish complex containing residual H_2_O_2_. Finally, the UV–vis absorption of this complex was measured at 405 nm by a microplate reader (Aebi 1984). Measurement of GPx activity: GPx catalyses the oxidation of glutathione by cumene hydroperoxide. In the presence of glutathione (GSSG), it is immediately converted to the reduced form by simultaneous oxidation of NADPH to NADP. GPx activity was measured by a change in absorbance (decrease in readings over 3 min) at 340 nm (Paglia and Valentine 1967; Prohaska et al. 1977; Kraus and Ganther 1980).

### 2.7 Histopathologic Investigation

After the feeding period, the harvested fish were necropsied. Following necropsy, gill, liver, muscle (dorsal muscle) and kidney tissues were sampled. The samples were placed in 10% buffered formalin for fixation. The sample tissues fixed in 10% buffered formalin were taken into cassettes after shrinking and trimming. The cassettes were washed in running water for 12 h. The washed tissues were transferred to an automatic tissue tracking device (Leica TP 1020), and the routine tissue tracking procedure was performed. Following this procedure, the samples were embedded in a paraffin block. After the paraffin blocks were cooled, 5 µm thick sections were taken with a microtome (Leica RM 2125 RT) and transferred to slides. The prepared slides were kept in an oven for one night. The tissues removed from the oven were cooled to room temperature and deparaffinized by xylol series (three different xylols for 5 min each). After deparaffinization, they were kept in 100%, 96%, 80% and 70% alcohol for 3 min each. This was followed by washing in tap water.

After washing, they were kept in a haematoxylin stain for 5 min. This was followed by washing in tap water. Following the washing process, acid‐alcohol immersion was performed one time and then washed in tap water. Subsequently, immersion in ammonia water was performed three times, and the tissues were washed in tap water. After this process, they were kept in eosin for 30 s. Then, after immersion in two different 96% and 100% alcohols, they were clarified in three different xylols for 5 min each. Finally, the sections removed from xylol were coverslipped with Entellan and made ready for examination. The sections were examined under a light microscope (Olympus BX51, Tokyo, Japan) and photographed (Olympus EP50, Tokyo, Japan).

Haematoxylin–eosin (H–E) staining of the gill, liver, muscle and kidney tissues was used to score the pathological findings on a scale of “(−) to 3” (−: *none*, 1: *mildly severe*, 2: *moderately severe* and 3: *severe*).

### 2.8 Chemical Composition of Fish Meat

The proximate composition of fish samples was analysed in triplicate using established methodologies. Lipid content was determined following the Bligh and Dyer (1959) method, while moisture and ash content were assessed using protocols outlined by AOAC (1990). Total crude protein content was measured using the Kjeldahl method, as described by AOAC (1984).

### 2.9 Statistical Analyses

Statistical analyses were performed using SPSS 18.0 (SPSS Inc., Chicago, IL, USA) to determine the differences between the groups in growth parameters and changes in blood parameters of fish fed with feed supplemented with anise oil at different rates.

## 3 Results

At the end of this research, the effects of Nile tilapia (*O. niloticus*) fed with special feeds added with different rates (0.00%, 0.10%, 0.20% and 0.30%) of anise (*Pimpinella anisum*) oil on growth performance, blood haematological parameters, biochemical parameters, oxidative stress parameters, histopathology and muscle nutrient content were investigated.

### 3.1 Growth Performance

In this study, 0.00%, 0.10%, 0.20% and 0.30% pure anise oil were added to Nile tilapia (*O. niloticus*) feed, and its effects on the growth performance of fish were investigated. In this study, fish with an average initial body weight of 74.20 ± 1.24 g were used, and at the end of the 40‐day feeding period, the fish reached a final weight of 114.43 ± 0.40 g, 121.14 ± 0.23 g, 125.79 ± 0.43 g and 121.36 ± 0.36 g, respectively (Table 1).

**TABLE 1:** Effects of dietary anise oil on growth performance of Nile tilapia.

	ANS 1	ANS 2	ANS 3	ANS 4
Initial weight	74.20 ± 1.24^a^	74.20 ± 1.24^a^	74.20 ± 1.24^a^	74.20 ± 1.24^a^
Final weight	114.43 ± 0.40^c^	121.14 ± 0.23^b^	125.79 ± 0.43^a^	121.36 ± 0.36^b^
Live weight gain	40.23 ± 0.40^c^	46.94 ± 0.22^b^	51.59 ± 0.42^a^	47.16 ± 0.49^b^
Feed conversion rate	1.87 ± 0.02^a^	1.75 ± 0.03^b^	1.67 ± 0.01^c^	1.74 ± 0.03^b^
Specific growth rate	1.08 ± 0.00^c^	1.23 ± 0.00^b^	1.32 ± 0.00^a^	1.23 ± 0.01^b^
Protein efficiency ratio	1.37 ± 0.01^c^	1.47 ± 0.02^b^	1.54 ± 0.01^a^	1.47 ± 0.02^b^
Feed consumption	75.31 ± 1.02^c^	82.10 ± 0.92^b^	86.08 ± 0.07^a^	82.11 ± 0.71^b^
Daily feed consumption	1.88 ± 0.03^c^	2.05 ± 0.02^b^	2.15 ± 0.01^a^	2.05 ± 0.02^b^

*Note*: ANS 1 (Control): 0.00% anise oil; ANS 2: 0.10% anise oil; ANS 3: 0.20% anise oil; ANS 4: 0.30% anise oil. There is a statistical difference between the data shown with different letters in the same row (*p *< 0.05).

### 3.2 Effects of Dietary Pure Anise Oil on Muscle Nutrient Content of Nile Tilapia

Although feed additives added to fish feeds have a positive effect on growth, the possible effects on muscle nutrient content should be known in order to recommend this substance as a good feed additive. One of the most important factors determining the flavour, cooking technique and even the shelf life of the fish is the fat content of the fish meat. In this study, the effects of pure anise oil added to fish feed on muscle nutrient content of Nile tilapia were determined, and the results are shown in Table 2. Pure anise oil added to fish feed increased the crude protein, lipid and crude ash content of Nile tilapia while decreasing the moisture content of the fish meat.

**TABLE 2:** Effects of dietary pure anise oil on muscle nutrient content of Nile tilapia.

	Muscle nutrient content			
Groups	Crude protein	Lipid	Crude ash	Moisture
ANS 1	17.65 ± 0.01^d^	1.19 ± 0.01^d^	1.21 ± 0.01^d^	79.15 ± 0.02^a^
ANS 2	17.97 ± 0.02^c^	1.27 ± 0.02^c^	1.33 ± 0.01^c^	78.51 ± 0.03^b^
ANS 3	18.19 ± 0.15^b^	1.49 ± 0.01^b^	1.42 ± 0.01^b^	78.03 ± 0.02^c^
ANS 4	19.04 ± 0.05^a^	1.62 ± 0.018^a^	1.53 ± 0.01^a^	77.21 ± 0.03^d^

*Note*: ANS 1 (Control): 0.00% anise oil; ANS 2: 0.10% anise oil; ANS 3: 0.20% anise oil; ANS 4: 0.30% anise oil. There is a statistical difference between the data shown with different letters in the same column (*p *< 0.05).

### 3.3 Effects of Dietary Anise Oil on Blood Parameters

In this study, some of the haematological parameters, which are important indicators of the health status of fish, were examined. These parameters are shown in Table 3, and when the values presented are analysed, it is seen that although there were no significant differences between the groups in some values, significant differences were found in some parameters (*p *> 0.05).

**TABLE 3:** Effect of dietary anise oil on blood parameters of Nile tilapia.

	ANS 1	ANS 2	ANS 3	ANS 4
RBC (m/mm^3^)	1.81 ± 0.02^c^	1.87 ± 0.02^b^	1.93 ± 0.01^a^	1.80 ± 0.02^c^
Hb (g/dl)	12.58 ± 0.35^c^	13.77 ± 0.43^b^	17.33 ± 0.37^a^	13.95 ± 0.31^b^
Hct (%)	41.24 ± 0.39^d^	48.10 ± 0.87^b^	50.60 ± 1.31^a^	43.39 ± 0.74^c^
MCV (fL)	228.46 ± 2.85^c^	257.23 ± 4.39^a^	262.64 ± 7.25^a^	240.42 ± 3.72^b^
MCH (pg)	69.72 ± 2.04^a^	73.62 ± 2.10^c^	89.97 ± 2.27^a^	77.29 ± 2.04^b^
MCHC (g/dL)	30.52 ± 0.84^c^	28.62 ± 0.65^d^	34.27 ± 0.93^a^	32.15 ± 0.94^b^

*Note*: ANS 1 (Control): 0.00% Anise oil; ANS 2: 0.10% Anise oil; ANS3: 0.20% Anise oil; ANS4: 0.30% Anise oil. There is a statistical difference between the data shown with different letters in the same row (*p *< 0.05).

Abbreviations: Hb, haemoglobin; Hct, haematocrit; MCH, mean red blood cell haemoglobin; MCHC, mean red blood cell haemoglobin concentration; MCV, mean red blood cell volume; RBC, erythrocyte.

### 3.4 Effects of Dietary Anise Oil on Blood Biochemistry Parameters of Nile Tilapia

Analysis of haematological, serum biochemical and enzymatic component levels provides useful information for the detection and diagnosis of metabolic disorders and diseases in fish. For this purpose, the blood biochemistry of the fish fed within the scope of the study was analysed, and the results are shown in Table 4.

**TABLE 4:** Effect of dietary anise oil on blood biochemistry of Nile tilapia.

	ANS 1	ANS 2	ANS 3	ANS 4
Cho (mg/dL)	125.80 ± 0.77^b^	110.12 ± 0.12^c^	102.46 ± 1.06^d^	140.69 ± 1.79^a^
Trg (mg/dL)	100.76 ± 1.41^b^	73.94 ± 1.60^c^	63.04 ± 0.66^d^	119.97 ± 1.42^a^
Glu (mg/dL)	41.28 ± 0.48^b^	35.78 ± 0.60^c^	33.19 ± 0.21^d^	54.74 ± 0.36^a^
ALP (U/L)	51.25 ± 1.35^b^	40.43 ± 0.55^c^	36.16 ± 0.95^d^	63.12 ± 0.15^a^
AST (U/L)	271.06 ± 1.89^b^	245.99 ± 0.63^c^	240.48 ± 0.72^d^	330.17 ± 2.65^a^
ALT (U/L)	25.59 ± 0.45^b^	20.46 ± 0.55^c^	18.79 ± 0.60^a^	32.54 ± 0.35^a^
TP (g/dL)	5.87 ± 0.09^b^	6.70 ± 0.20^a^	6.92 ± 0.06^a^	5.06 ± 0.05^d^

*Note*: ANS 1 (Control): 0.00% anise oil; ANS 2: 0.10% anise oil; ANS 3: 0.20% anise oil; ANS 4: 0.30% anise oil. There is a statistical difference between the data shown with different letters in the same row (*p *< 0.05).

Abbreviations: ALP, alkaline phosphatase; ALT, alanine aminotransferase; AST, aspartate aminotransferase; Cho, cholesterol; Glu, glucose; TP, total protein; TRG, triglyceride.

### 3.5 Effects of Dietary Anise Oil on Oxidative Stress Parameters

In this study, oxidative stress parameters were determined from blood serum samples of fish, and the results are presented in Table 5. TAS, TOS, OSI, CAT, SOD, MDA and Gpx were determined from sera obtained from blood samples taken from fish. It was determined that pure anise oil added up to 0.20% to the fish feed caused an increase in serum TAS, TOS, CAT, SOD and GPx levels of Nile tilapia, and when anise oil was added at a higher rate, it caused a decrease. In addition, pure anise oil added to the fish feed at 0.10% and 0.20% decreased the serum MDA value of Nile tilapia, while pure anise oil added at 0.30% increased the MDA value.

**TABLE 5:** Effects of dietary pure anise oil on oxidative stress parameters of Nile tilapia.

Oxidative stress parameters				
	ANS 1‐control	ANS 2	ANS 3	ANS 4
TAS (mmol/L)	2.05 ± 0.02^c^	2.18 ± 0.01^b^	2.34 ± 0.02^a^	2.01 ± 0.01^d^
TOS (µmol/L)	19.49 ± 0.78^d^	42.72 ± 1.37^b^	65.42 ± 1.12^a^	27.98 ± 0.68^c^
OSI	0.95 ± 0.03^d^	1.96 ± 0.06^b^	2.80 ± 0.07^a^	1.39 ± 0.02^c^
CAT (U/mL)	207.04 ± 1.58^c^	225.02 ± 0.83^b^	237.52 ± 1.42^a^	193.53 ± 0.75^d^
SOD (U/mL)	431.80 ± 2.05^c^	464.67 ± 2.68^b^	488.32 ± 1.17^a^	375.50 ± 1.63^d^
MDA (mmol/L)	98.79 ± 0.57^b^	92.30 ± 0.38^c^	82.96 ± 0.27^d^	106.04 ± 0.46^a^
GPx (U/mL)	192.14 ± 0.58^c^	212.98 ± 0.76^b^	241.62 ± 1.27^a^	191.01 ± 1.33^d^

*Note*: ANS 1 (Control): 0.00% anise oil; ANS 2: 0.10% anise oil; ANS 3: 0.20% anise oil; ANS 4: 0.30% anise oil. There is a statistical difference between the data shown with different letters in the same row (*p *< 0.05).

Abbreviations: CAT, catalase; GPx, glutathione peroxidase; MDA, malondialdehyde; SOD, superoxide dismutase; TAS, total antioxidant status; TOS, total oxidant status.

TAS in the control group was 2.05 mmol/L. It was determined that anise oil added to the diet changed the TAS levels in Nile tilapia blood, and this value ranged between 2.01 and 2.34 mmol/L in the treatment groups. Anise oil added up to 0.20% to the fish feed increased the TOS of Nile tilapia, and anise oil added at higher doses decreased TOS, and there was a statistical difference between the study groups and the control group (*p* < 0.05). The results were calculated as 19.49 ± 0.78 µmol/L, 42.72 ± 1.37 µmol/L, 65.42 ± 1.12 µmol/L and 27.98 ± 0.68 µmol/L for ANS1, ANS2, ANS3 and ANS4, respectively.

### 3.6 Histopathologic Findings

After the feeding period, gills, liver, muscle and kidney were harvested from the harvested fish, and histopathological findings were scored, and the results are presented in Table 6.

**TABLE 6:** Number and scores of histopathological findings observed in organs.

		Gill			Liver			Muscle			Kidney	
Groups	Lesion scores	Oedema in lamellae	Hyperaemia	Inflammatory cell infiltration	Hydropic degeneration	Hyperaemia	Necrosis in hepatocytes	Atrophy	Inflammatory cell infiltration	Necrosis	Degeneration of tubular epithelium	Necrosis of tubular epithelium
ANS‐1 group (*N* = 30)	−	30	28	30	29	29	30	30	30	30	30	30
	+1	—	2	—	1	1	—	—	—	—	—	—
	+2	—	—	—	—	—	—	—	—	—	—	—
	+3	—	—	—	—	—	—	—	—	—	—	—
	Total	**—**	**2**	**—**	**1**	**1**	**—**	**—**	**—**	**—**	**—**	**—**
ANS‐2 group (*N* = 30)	−	28	27	30	28	27	30	27	30	30	28	30
	+1	2	3	—	2	3	—	3	—	—	2	—
	+2	—	—	—	—	—	—	—	—	—	—	—
	+3	—	—	—	—	—	—	—	—	—	—	—
	Total	**2**	**3**	**—**	**2**	**3**	**—**	**3**	**—**	**—**	**2**	**—**
ANS‐3 group (*N* = 30)	−	28	28	30	29	29	30	30	29	30	29	30
	+1	2	2	—	1	1	—	—	1	—	1	—
	+2	—	—	—	—	—	—	—	—	—	—	—
	+3	—	—	—	—	—	—	—	—	—	—	—
	Total	**2**	**2**	**—**	**1**	**1**	**—**	**—**	**1**	**—**	**1**	**—**
ANS‐4 group (*N* = 30)	−	25	26	28	26	25	28	25	27	28	27	27
	+1	3	1	2	2	3	1	4	2	1	1	2
	+2	2	3	—	2	2	1	1	1	1	2	1
	+3	—	—	—	—	—	—	—	—	—	—	—
	Total	**5**	**4**	**2**	**4**	**5**	**2**	**5**	**3**	**2**	**3**	**3**

Abbreviations: (−), none; (+1), mildly severe; (+2), moderately severe; (+3), severe.

The bold values represent the total number of fish in each group showing the respective lesion, summing the occurrences across all severity levels (+1 to +3).

**FIGURE 1:**
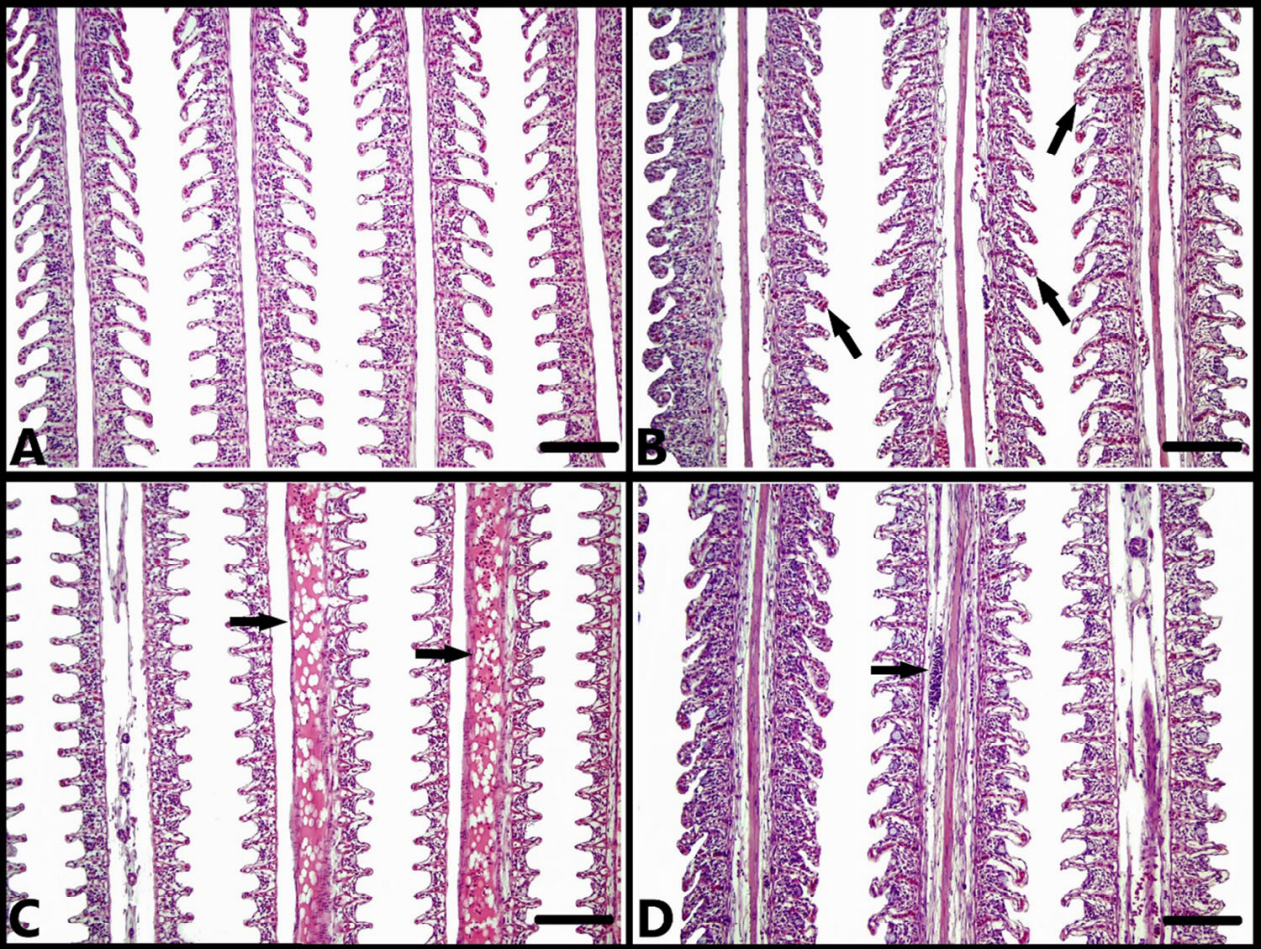
Microscopic examination of gill sections. (A) Normal histological appearance of gills, control group (ANS‐1), H–E staining, bar: 100 µm. (B) hyperaemia of secondary lamellae (arrows), ANS‐2 group, H–E staining, bar: 100 µm. (C) Hyperaemia and oedema in primary lamellae (arrows), ANS‐3 group, H–E staining, bar: 100 µm. (D) Hyperaemia in primary and secondary lamellae, hyperplasia in lamellae and inflammatory cell infiltration in primary lamellae (arrow), ANS‐4 group, H–E staining, bar: 100 µm.

In the histopathological examination of the gills, no significant histopathological findings were observed in the fish in the control (ANS‐1) group (Figure 1A). Mild secondary lamellar hyperaemia was observed in the fish in the 0.10% (ANS‐2) group (Figure 1B), while primary lamellar oedema was detected in the 0.20% (ANS‐3) group (Figure 1C). In the 0.30% anise oil (ANS‐4) group, mild inflammatory cell infiltration, moderate primary and secondary lamellar hyperaemia, and oedema were observed in the primary lamellae (Figure 1D).

**FIGURE 2:**
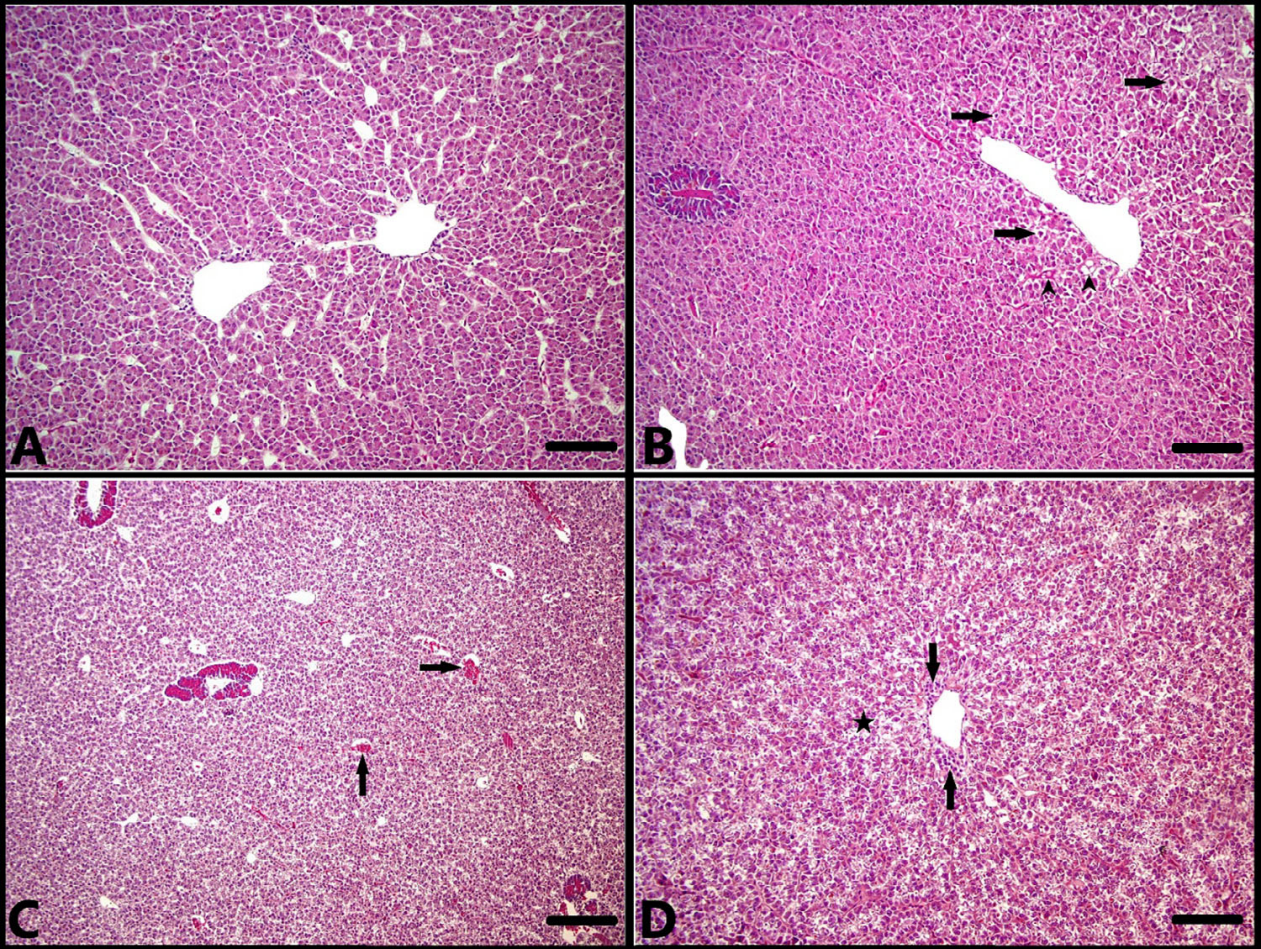
Microscopic examination of liver sections. (A) Normal histologic appearance of the liver, control group (ANS‐1), H–E staining, bar: 100 µm. (B) Mild hydropic degeneration of hepatocytes (arrows) and oedema in the disseal spaces (arrowheads), ANS‐2 group, H–E staining, bar: 100 µm. (C) Hyperaemia in the vena centralis (arrows), ANS‐3 group, H–E staining, bar: 200 µm. (D) Mild necrosis and moderate hydropic degeneration of hepatocytes (asterisk), oedema in the interspaces of the disse, and mild inflammatory cell infiltration around the vena centralis (arrows), ANS‐4 group, H–E staining, bar: 100 µm.

Histopathologic examination of the liver sections revealed no significant histopathologic findings in the ANS‐1 fish (Figure 2A). Mild hydropic degeneration and hyperaemia were observed in the ANS‐2 and ANS‐3 groups (Figure 2B,C). In the ANS‐4 group, moderate hydropic degeneration, hyperaemia, necrosis in hepatocytes and mild inflammatory cell infiltration around the vena centralis were determined (Figure 2D). When the ANS‐4 group was compared with the other groups, it was observed that the damage in the liver of the fish in the ANS‐4 group was more severe.

**FIGURE 3:**
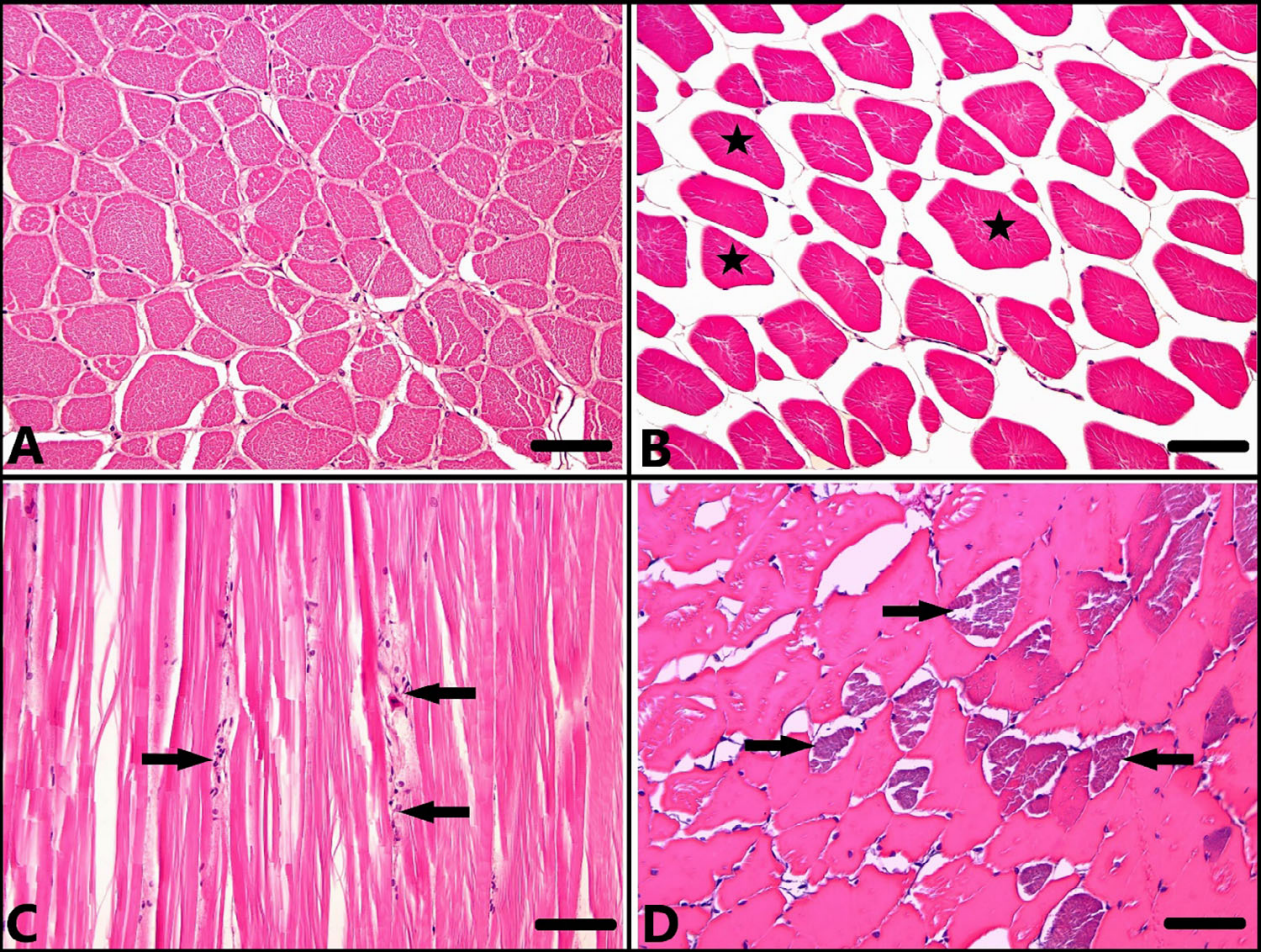
Microscopic examination of muscle tissue sections. (A) Normal histologic appearance of muscle tissue, control group (ANS‐1), H–E staining, bar: 50 µm. (B) Muscle atrophy (asterisks), ANS‐2 group, H–E staining, bar: 50 µm. (C) Inflammatory cell infiltration between muscle bundles (arrows), ANS‐3 group, H–E staining, bar: 50 µm. (D) Atrophy and necrosis of muscles (arrows), ANS‐4 group, H–E staining, bar: 50 µm.

In the histopathologic examination of the dorsal muscle tissue, no histopathologic findings were observed in the ANS‐1 (Figure 3A), while mild atrophy was observed in some of the fish in the ANS‐2 group (Figure 3B). While no muscle atrophy was observed in the ANS‐3 group, inflammatory cell infiltration between muscle bundles was observed in only one fish (Figure 3C). In the ANS‐4 group, muscle atrophy, inflammatory cell infiltration and muscle necrosis were observed (Figure 3D). When the muscle tissues of the fish in the ANS‐3 group were compared with the other groups, it was noted that the histopathologic changes seen in the muscle tissues were rare.

**FIGURE 4:**
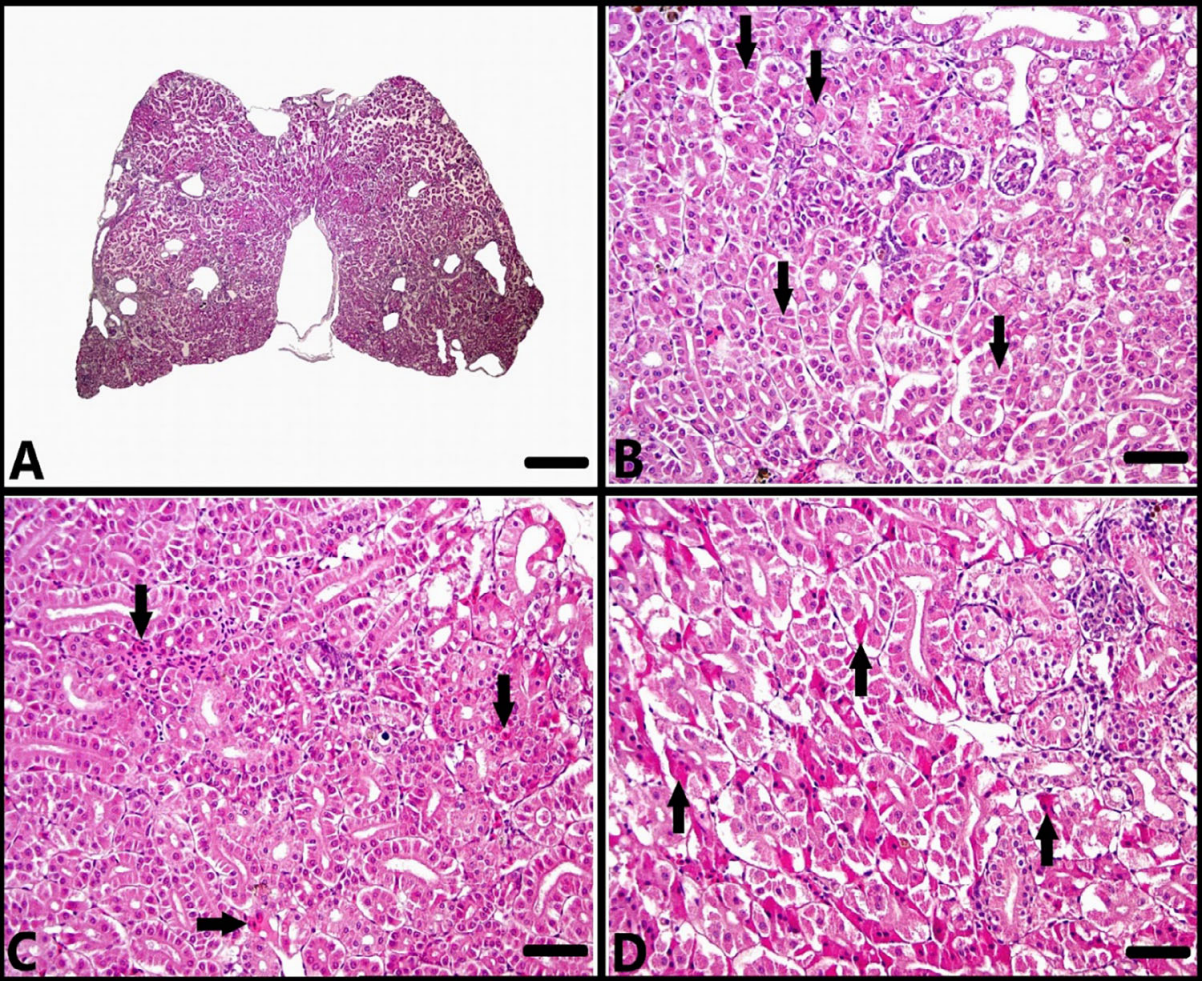
Microscopic examination of kidney sections. (A) Normal histological appearance of the kidney, control group (ANS‐1), H–E staining, bar: 500 µm. (B) Mild degeneration of tubular epithelium (arrows), ANS‐2 group, H–E staining, bar: 50 µm. (C) Mild haemorrhage in the interstitium (arrows), ANS‐3 group, H–E staining, bar: 50 µm. (D) Moderate degeneration and necrosis of tubule epithelium (arrows), ANS‐4 group, H–E staining, bar: 50 µm.

In the histopathological examination of the kidney sections, while no histopathological findings were observed in the ANS‐1 (Figure 4A), some of the fish in the ANS‐2 and ANS‐3 groups showed mild degeneration of tubular epithelium and mild haemorrhage in the interstitium (Figure 4B,C). In some of the fish in the ANS‐4 group, moderate degeneration and necrosis of tubular epithelium and moderate haemorrhage in the interstitium were observed (Figure 4D).

## 4 Discussion

The two most common measures of responses to a particular diet or ingredient in fish are growth performance and feed utilization. Growth is measured as a function of specific nutrient gain (e.g., protein), weight gain or length. One objective of aquaculture is to maximize production in terms of biomass. Therefore, body weight gain is a common measure of performance (Lazo and Davis 2000).

The study demonstrates the dose‐dependent effect of anise oil supplementation on the growth performance of Nile tilapia (*O. niloticus*). Our findings indicate that 0.10%–0.20% anise oil supplementation significantly enhances growth, whereas 0.30% may negatively impact growth performance. These findings highlight the functional benefits of anise oil as a feed additive and emphasize the need for optimal dosage determination in aquaculture. In the study, a significant increase in growth performance was observed in the groups fed with 0.10% and 0.20% anise oil, while a decrease in growth performance was observed in the group fed with 0.30% anise oil. The increase in growth performance observed in the groups fed with 0.10% and 0.20% anise oil is thought to be due to the beneficial properties of anise oil, which contains bioactive compounds such as antioxidants and essential oils known to support metabolic efficiency and feed utilization.

Although there are not many studies examining the effect of anise on the growth parameters of fish, there are many studies on broilers and pigs. In a study conducted by adding 0.1%, 0.5%, 1.5%, 3% and 5% aniseed to rainbow trout feeds, the live weight gains of the fish were calculated at the end of 8 weeks, and similar to the results of the current research, it was observed that growth performance increased at low doses, but live weight gain decreased after 1.5% (Çelik 2021). In another study, it was reported that broilers fed with diets supplemented with anise oil at different rates showed higher growth performance than the control group (Charal et al. 2017).

The findings of the present study on the dose‐dependent effects of anise oil supplementation in Nile tilapia (*O. niloticus*) are in line with existing studies on the use of essential oils in aquaculture. In particular, a study by Hussain et al. (2023) investigated the dietary effects of anise essential oil on Nile tilapia and found that supplementation improved growth performance and reduced feed conversion rates compared to the control group.

However, the decline in growth performance observed in the group fed pure anise oil feed supplemented at 0.30% in your study suggests that there is beyond a threshold which anise oil may exert adverse effects. This phenomenon is consistent with the findings reported by Bandeira Junior et al. (2022), who stated that higher doses of essential oils do not always improve growth and may impair the physiological state of fish.

A study by Elkomy et al. (2023) showed that dietary supplementation with 0.5% anise oil improved growth performance, immune response and antioxidant activity in quail. The study attributed these effects to the presence of bioactive compounds in anise oil, including trans‐anethole, which has antioxidant and antimicrobial properties.

The addition of pure anise oil to fish feed affected the nutritional composition of Nile tilapia (*O. niloticus*) meat. The observed increase in crude protein, lipid and ash content, together with a decrease in moisture content, emphasize the multifaceted effects of dietary anise oil supplementation on fish metabolism and tissue composition. The increase in crude protein content of fish meat can be attributed to improved feed conversion efficiency and nitrogen retention facilitated by anise oil supplementation.

Bioactive compounds such as anethole in anise oil may stimulate the secretion of digestive enzymes, leading to improved protein digestion and assimilation (Hussain et al. 2023). In fish, the activity of digestive enzymes is closely linked to both diet composition and the physiological development of the gastrointestinal tract. For example, Infante and Cahu (2007) reported that pancreatic enzyme expression, including trypsin and lipase, could be modulated by dietary protein and lipid content through hormonal pathways such as cholecystokinin signalling, particularly in early life stages. Additionally, Hidalgo et al. (1999) demonstrated that omnivorous fish such as common carp exhibit significantly higher amylase and protease activities in the digestive tract compared to carnivorous species, indicating a greater enzymatic capacity for the breakdown of carbohydrates and proteins. This enzymatic plasticity, when influenced by bioactive feed additives like anise oil, may provide a mechanistic basis for improved feed efficiency and growth performance in Nile tilapia.

The antioxidant properties of anise oil may reduce oxidative stress and thus enable more energy to be directed towards protein synthesis rather than stress responses. Furthermore, the bioactive compounds of anise oil may influence lipid metabolism by promoting lipid accumulation in muscle tissues while maintaining overall energy balance. With improved digestion, a higher proportion of dietary lipids may be available for accumulation rather than being metabolized for energy.

The increase in crude ash content is probably due to the positive effect of essential oils on intestinal health and improved absorption of dietary minerals such as calcium and phosphorus (Bandeira Junior et al. 2022).

In this study, it was found that the muscle moisture content of fish fed with anise oil decreased. Reducing moisture content in fish meat is beneficial for improving its quality, as lower moisture levels are associated with improved texture, extended shelf life and reduced microbial growth. High moisture content in fish facilitates microbial activity and leads to rapid spoilage. By reducing moisture levels, water activity is reduced, thereby inhibiting enzymatic reactions that cause microbial proliferation and spoilage (Tavares et al. 2021). In conclusion, the inclusion of pure anise oil in Nile tilapia feed significantly improves the nutritional and quality characteristics of fish meat by reducing moisture while increasing crude protein, lipid and ash content. These changes highlight the potential of anise oil as a functional feed additive in sustainable aquaculture practices.

The observed effects of anise oil supplementation on the haematological parameters of Nile tilapia (*O. niloticus*) reflect its dose‐dependent effect on fish health and physiological functions. The increase in RBC (red blood cell count), Hb concentration and Hct levels with the addition of 0.10% and 0.20% anise oil indicates improved haematological status, while the decrease at 0.30% indicates a potentially toxic or stressful effect at higher doses. These findings are crucial for understanding the role of anise oil as a functional feed additive in aquaculture.

The improvement in haematological parameters in the 0.10% and 0.20% groups observed in the study indicates that anise oil at these doses positively affects the health and oxygen‐carrying capacity of Nile tilapia. Anise oil contains bioactive compounds such as anethole, which shows strong antioxidant properties. These compounds may reduce oxidative stress, leading to healthier erythropoiesis (RBC production) and improved overall haematological function (Hussain et al. 2023).

The immunostimulatory effects of some oils, such as anise oil, may increase the ability of fish to produce RBCs and maintain higher Hb levels, thus improving resistance to stress and disease (Bandeira Junior et al. 2022).

In addition, the decrease in haematological parameters observed in the group fed 0.30% anise oil‐supplemented feed suggests that higher doses may have a negative effect. Anise oil given in high concentrations can become toxic, impair erythropoiesis and cause haemolysis (destruction of RBCs). This can result in lower RBC and Hb levels and a decrease in Hct (Fernández‐Mendez et al. 2024).

The findings of the study show that anise oil supplementation at 0.10% and 0.20% levels decreased serum Cho, Trg and Glu in Nile tilapia (*O. niloticus*), while a 0.30% supplementation increased these parameters. This suggests that anise oil has a dose‐dependent effect on the metabolic health of Nile tilapia.

At lower supplementation levels, bioactive compounds in anise oil, such as anethole, may increase lipid and Glu metabolism, resulting in decreased serum Cho, Trg and Glu levels. These results are consistent with studies reporting that some aromatic plant oils can improve growth performance and health status in Nile tilapia (Ahmed et al. 2022; Öz and Dikel 2022; Esen 2024). Bioactive compounds in anise oil, particularly trans‐anethole, may enhance lipid and Glu metabolism. Although evidence in fish is limited, studies in mammalian models have shown that trans‐anethole can reduce hepatic lipid accumulation and improve Glu handling by modulating key metabolic enzymes (Song et al. 2020). Similar regulatory mechanisms have also been suggested in fish species (Fang et al. 2024). Therefore, anise oil may contribute to improved energy utilization in Nile tilapia. However, the elevated lipid and Glu levels observed at the 0.30% inclusion level may indicate impaired metabolic regulation, possibly due to the pro‐oxidant effects of excessive bioactive intake.

Notably, the increase in these parameters at the 0.30% supplementation level suggests that higher doses may impair metabolic processes, potentially due to the pro‐oxidant effects of excess bioactive compounds. This is consistent with the observations of Li et al. (2023), who found that high Cho intake impaired liver function and reduced mitochondrial numbers in Nile tilapia.

The findings of the study show that supplementation of Nile tilapia (*O. niloticus*) diets with up to 0.20% pure anise oil increases serum TAS and activities of key antioxidant enzymes, including CAT, SOD and GPx. However, supplementation beyond this concentration results in a decrease in these antioxidant parameters.

The increase in TAS and antioxidant enzyme activities observed at lower anise oil concentrations suggests that bioactive compounds such as anethole may enhance the antioxidant defence mechanisms of fish. This enhancement may involve up‐regulation of antioxidant enzyme expression or direct scavenging of reactive oxygen species (ROS), thereby reducing oxidative stress. Similar effects have been observed in studies with other essential oils. For example, dietary supplementation with basil essential oil was reported to improve the antioxidant capacity of Nile tilapia with increased activities of CAT, SOD and GPx (Mansour et al. 2023). In summary, anise oil supplementation up to 0.20% may enhance the antioxidant defence system in Nile tilapia, while higher concentrations may have detrimental effects. These findings highlight the need for precise dosage optimization when adding anise oil to fish diets to promote fish health and prevent oxidative stress‐related damage.

In the study conducted by Metin et al. (2024), the histopathological effects of different concentrations of anise oil on carp (*Cyprinus carpio* L. 1758) were examined. They reported hyperaemia, intense inflammatory cell infiltration in the gills and mild inflammatory cell infiltration in the liver and muscles. Similarly, the present study observed severe hyperaemia and intense inflammatory cell infiltration in the gills; mild necrosis and inflammatory cell infiltration in the liver; atrophy, necrosis and inflammatory cell infiltration in the muscles; and moderate tubular degeneration and necrosis in the kidneys of fish fed with 0.30% pure anise oil‐supplemented feed. These findings underscore a dose‐dependent toxicopathological effect of anise oil.

At lower concentrations (0.10% and 0.20%), the addition of pure anise oil resulted in only mild histopathological findings, suggesting that it may be used as a feed additive without severe adverse effects at these levels. However, the toxic effects observed at the 0.30% concentration indicate that excessive supplementation of anise oil can disrupt normal physiological functions and negatively impact fish health. The observed growth performance decline and feed utilization inefficiency in the ANS‐4 group further support this conclusion.

The toxic effects of high doses of anise oil are likely related to its bioactive compounds, such as anethole, which may exert pro‐oxidant or cytotoxic effects when administered in excessive amounts. These findings align with previous studies on the toxicity of essential oils at higher doses, which emphasize the importance of careful dose optimization.

The histopathological findings in this study have significant implications for aquaculture feed formulation. While low concentrations of anise oil may provide benefits such as antimicrobial or antioxidant properties, higher concentrations pose a clear risk to fish health. Future studies should explore the long‐term effects of anise oil supplementation, its impact on immune responses, and its interaction with other feed components. Additionally, investigating alternative delivery methods or encapsulation technologies may help mitigate its toxic effects while maintaining its potential benefits.

In conclusion, this study demonstrates that while low doses of pure anise oil may be safe and beneficial, excessive supplementation can lead to significant toxicopathological effects. These findings highlight the need for caution in determining the optimal inclusion level of anise oil in fish diets to maximize its benefits while minimizing risks.

## 5 Conclusion

This study demonstrates that dietary supplementation with 0.10% and 0.20% anise oil significantly enhances growth performance, feed efficiency, antioxidant status and haematological parameters in Nile tilapia (*O. niloticus*). However, supplementation at 0.30% resulted in metabolic inefficiencies, oxidative stress and mild histopathological changes, indicating a potential toxicity threshold. These findings provide strong evidence that anise oil could serve as a natural alternative to synthetic growth promoters in aquaculture. The antioxidant and immunostimulatory effects observed in this study suggest that anise oil supplementation may play a vital role in improving fish health, disease resistance and overall sustainability in intensive farming systems. Additionally, the observed changes in muscle composition indicate that anise oil may influence fish meat quality, highlighting its potential impact on both nutritional and commercial value.

Despite these promising results, further research is needed to determine the long‐term effects of anise oil supplementation across different growth stages and production environments. Future studies should also focus on elucidating the molecular mechanisms underlying its antioxidant and immunostimulatory properties, as well as evaluating its effectiveness in other commercially important fish species. Additionally, optimizing formulation strategies, such as microencapsulation, could enhance its bioavailability while mitigating potential toxicity risks. Given the increasing demand for sustainable and eco‐friendly aquaculture practices, integrating plant‐derived bioactive compounds like anise oil into commercial fish feeds holds significant promise. Large‐scale farm trials, cost‐effectiveness assessments and regulatory evaluations will be essential to facilitate its widespread adoption in the industry. By advancing our understanding of the interactions between phytogenic feed additives and fish physiology, this study contributes to ongoing efforts to enhance aquaculture productivity while minimizing environmental impact.

One of the main limitations of the present study is the lack of compositional analysis of the anise oil used. Future research should include detailed chemical characterization, such as GC‐MS profiling, to identify and quantify the specific bioactive compounds responsible for the observed physiological and biochemical effects in fish.

## Author Contributions

**Serkan TOK**: investigation, conceptualization, writing – original draft. **Mustafa ÖZ**: investigation, writing – original draft, writing – review and editing, methodology, formal analysis, data curation, supervision, project administration. **Suat DIKEL**: formal analysis, investigation, writing – original draft, writing – review and editing, methodology.

## Conflicts of Interest

The authors declare no conflicts of interest.

### Peer Review

The peer review history for this article is available at https://publons.com/publon/10.1002/vms3.70443.

## Data Availability

The data that support the findings of this study are available on request from the corresponding author. The data are not publicly available due to privacy or ethical restrictions.
